# Supply of Lato Sensu Postgraduate courses in medicine in Brazil

**DOI:** 10.1590/1806-9282.20251052

**Published:** 2025-10-27

**Authors:** Ivan Wilson Hossni Dias, José Eduardo Lutaif Dolci, Cristiane de Jesus Almeida, Mário César Scheffer

**Affiliations:** 1 Universidade de São Paulo, Faculty of Medicine, Department of Preventive Medicine – São Paulo (SP), Brazil.; 2 Universidade de São Paulo, Faculty of Medicine, Brazilian Medical Demographics Research Group – São Paulo (SP), Brazil.; 3 Associação Médica Brasileira, Department of Scientific – São Paulo (SP), Brazil.

**Keywords:** Specialization, Distance education, Physicians, Medical faculty, Private sector

## Abstract

**OBJECTIVE:**

Since a significant number of professionals in Brazil did not hold a specialist title, the aim of this study was to describe and analyze the supply of Lato Sensu Postgraduate courses in medicine.

**METHODS:**

Lato Sensu Postgraduate courses offered between February and May 2024 were surveyed using the e-MEC platform (Ministry of Education) and the websites of the offering institutions. The variables considered were medical specialty, number of places, workload, price, teaching modality, and public or private nature. Descriptive analyses and statistical tests of association were performed.

**RESULTS:**

A total of 2,148 Lato Sensu Postgraduate courses aimed at doctors were identified and offered by 373 institutions, the majority (60.7%) of which were located in the Southeast, with half having a total workload of up to 420 h. The average duration was 13.3±7.4 months; there were 30.6±59.6 vacancies on offer, and the average total price was R$15,782.44±35,372.88. Approximately 90% were paid for, and 41.2% were distance-learning courses, the latter of which had a shorter duration, lower cost, and more places than other types of education.

**CONCLUSION:**

Lato Sensu Postgraduate courses in medicine in Brazil reflect a heterogeneous, predominantly private universe with great variation in the characteristics of the courses on offer. This dispersion indicates the need for better regulation of Lato Sensu Postgraduate supply and marketing in Brazil, and in this way, the current study has important implications for education improvement.

## INTRODUCTION

In Brazil, legislation states that the title of specialist doctor can only be obtained following the completion of Medical Residency (MR) programs accredited by the National Medical Residency Commission/Ministry of Education (CNRM/MEC) or through specialty societies affiliated with the Brazilian Medical Association (AMB)^1,2^.

As of 2024, more than 244,000 doctors, approximately 40% of all practicing professionals in Brazil, did not hold a specialist title^3^.

Lato Sensu Postgraduate (LSPG) courses are defined by the MEC as continuing education programs aimed at those who have already graduated^4^. In medicine, doctors who hold only an LSPG certificate cannot advertise themselves as specialists^5^.

With a minimum workload of 360 h, LSPG courses can be offered by teaching and research institutions or entities related to the world of work^4^.

Private education groups have started to market LSPG courses on a larger scale, the provision of which does not require authorization or recognition from government bodies^6^.

This study aimed to describe and analyze the supply of LSPG courses in medicine in Brazil.

## METHODS

This was a cross-sectional observational study, with data on LSPG courses in medicine collected from February 11, 2024, to May 13, 2024. The initial data source was the e-MEC database, the official system of MEC. This database gathers information on higher education institutions, regulatory processes, evaluations of undergraduate programs, and declaratory information of LSPG courses.

In the "Specialization Course" field of e-MEC, the major area of "Health and Wellness" was selected. A total of 117 search words or expressions were defined, both generic—such as "medicine," "doctor," "health"—and specific, corresponding to the 55 medical specialties and 62 areas of medical practice recognized in Brazil^7^.

This resulted in a list of 985 institutions offering 10,267 LSPG courses. We then moved on to the second stage of the study, which aimed to collect primary data while also validating and supplementing the information on the e-MEC platform.

Based on the list of 985 institutions, the fieldwork involved five researchers. An electronic form (website) was used to extract data on the LSPG courses offered at the time of the survey.

The study included LSPG courses offered by registered institutions, but not reported in e-MEC, as well as those registered in e-MEC and actually offered.

Institutions that, unlike those registered on e-MEC, no longer offered courses were disregarded, as were courses that were reported in the e-MEC database at some point but were no longer being offered.

The institutions offering LSPG courses were analyzed according to whether they were public or private and their location (Federal Unit where they were based).

To characterize the LSPG courses, the following variables were considered: (a) medical specialty, area, or field of knowledge; (b) number of vacancies; (c) teaching modality (presential, distance learning, hybrid, or semi-presential); (d) duration (in months); (e) workload (in hours); (f) nature of the institution (public or private); and (g) pricing (total price of the course).

The public or private nature of the institution refers to its juridic nature^8^, while the public or private nature of the course refers to whether it is free or paid for.

Statistical analyses were performed using the STATA statistical package version 18.0. The adequacy of the parameters to the normal distribution was assessed using the Shapiro-Wilk test. Alongside descriptive statistical methods (mean, standard deviation, median, and frequency), the Kruskal-Wallis test was used to compare the teaching methods in cases where the quantitative variables did not follow a normal distribution. Dunn's post-hoc test with Bonferroni correction was performed to identify the modality responsible for the difference observed^9^.

The study was approved by the Research Ethics Committee (CEP/FMUSP) under CAEE Opinion No. 71626323.8.0000.0068, dated August 11, 2023.

## RESULTS

The study included 2,148 LSPG courses exclusively aimed at doctors and offered by 373 institutions.

Of these, 1,304 (60.7%) courses were offered in the Southeast, 704 (32.8%) in São Paulo, and 352 (16.4%) in Minas Gerais. There were 1,163 courses (54.1%) in the capital cities (Table 1).

**Table 1:** Absolute and relative frequencies of Lato Sensu Postgraduate courses in medicine according to selected variables, Brazil, 2024.

Variables	Categories	n	%
Location (type of municipality) (n=2,148)	Capital	1.163	54.14
	Others	985	45.86
Location (Greater Region) (n=2,148)	Southeast	1.304	60.71
	Northeast	257	11.96
	South	254	11.82
	Central-West	198	9.22
	North	135	6.28
Modalities of teaching (n=1,943)^a^	Distance learning	800	41.17
	Presential	927	47.71
	Semi-presential	216	11.12
Tuition (n=878)^b^	Free	84	9.57
	Paid	794	90.43
Nature of the institution (n=373)	Public	344	92.23
	Private	29	7.77
Offered by institutions that run a medical school (n=373)	No	257	68.90
	Yes	116	31.10

a Missing <10%;

b Missing >15%.

Of the courses whose modality was identified (n=1,953), 800 (41.2%) were offered in the distance-learning format and 927 (47.7%) were offered face-to-face. Approximately 90.4% of the courses were paid courses marketed predominantly by private institutions (92.2%).

The mean duration of the courses was 13.30 months. In terms of workload, 272 courses (12.7%) demanded less than 360 h, meaning they did not meet the minimum workload required by law. Alongside the workload, the number of places and the cost varied significantly (Table 2).

**Table 2:** Characteristics of Lato Sensu Postgraduate courses in medicine according to workload, duration, number of places, and value, Brazil, 2024.

Variables	n	Mean	Standard deviation	Median
Workload^a^	1,630	507.63	324.64	420.00
Duration^a^	1,660	13.30	7.38	12.00
Vacancies^b^	345	30.59	59.63	6.00
Pricing^b^	678	15,782.36	35,372.88	2,800.00

a Missing <10%;

b Missing >15%.

The specialties with the highest number of LSPG courses were Endocrinology and Metabolism (147 courses), Dermatology (129), Psychiatry (115), Radiology and Diagnostic Imaging (103), and Hematology and Hemotherapy (101).

Of the 373 institutions studied, 231 were located in the southern and southeastern regions. Approximately one-third (116 institutions) had undergraduate medical education. A total of 344 institutions were private, either for-profit or non-profit, and together offered 1,774 courses (82.6%); 29 institutions were public and together offered 374 courses (17.4%).

All characteristics of the courses on offer were associated with teaching type (p<0.01). There was a significant difference between distance-learning and present-day courses in terms of duration, number of places on offer, and course fees. There was a significant difference in workload between semi-presential courses and those offered by other modalities (Table 3).

**Table 3:** Characteristics of Lato Sensu Postgraduate courses according to teaching modality, Brazil, 2024 (n=1,943).

	EaD (n=800)	Presential (n=927)	Semi-presential (n=216)	p-value*
Workload (hours)	480 (360–720)	420 (360–680)	360 (360–430)	**<0.01**
Duration (months)	10 (6–12)	12 (12–24)	12 (12–18)	**<0.01**
Vacancies	50 (11–110)	4 (2–20)	20 (20–30)	**<0.01**
Pricing (R$)	2,162 (1,782–3,887)	8,400 (450–33,640)	20,000 (10,275–39,486)	**<0.01**

Data are presented as median (25–75 percentiles). EaD: distance learning.

* Statistically significant values from the Kruskal-Wallis test are indicated in bold.

## DISCUSSION OF THE RESULTS

The large number of LSPG courses identified in this study reveals a phenomenon that could be on the rise. As of 2024, more than 240,000 doctors in Brazil did not hold a specialist title^3^, and hence there could be a potential audience for LSPG courses.

There has been growing judicialization, with decisions that may be favorable toward^10^, but are mainly against^11,12^, authorizing doctors who hold an LSPG certificate to advertise themselves as specialists. The National Education Council (CNE), MEC, and CNRM have clarified that LSPG courses are not equivalent to medical residency^13^.

Notably, many of the LSPG courses were in the distance-learning format (approximately 40% of the courses). Although digital technologies allow for remote activities suited to the profiles or stages of certain courses^14,15^, the lack of practical content in a face-to-face environment is incompatible with good medical training in most areas^16,17^.

The offering of LSPG courses in medicine is concentrated both geographically, in the capitals and major centers, and economically, in private education business groups. Our study showed that approximately one-third of the institutions offered both undergraduate medical degrees and LSPG courses. It can be assumed that these institutions, most of which are private, seek to market the medical pathway from undergraduate to specialized medical training.

The wide range of courses offered across different medical specialties, along with considerable variation in location, format, workload, vacancies, duration, and cost, not only reflects the diversity of topics and objectives aimed by these programs but also underscores the fragility of the regulatory legislation of LSPG courses in Brazil.

Few LSPG courses are free or have an explicit relationship with the policies, programs, and goals of the Unified Health System (SUS). There are LSPG courses run by renowned institutions or accepted by specialty societies when scoring qualifications, but many others are offered by institutions supposedly with less experience and capacity in the area of the course.

This study revealed that nearly one-third of the institutions also offered undergraduate medical programs and LSPG courses. It can be assumed that these institutions, mostly private, seek to commercialize the medical career, from initial training to professional specialization.

This study has several limitations. First, the survey of different databases only partly explained the different missing values, depending on the variable studied. Second, given the multiple nomenclatures and descriptions of LSPG courses, approximations were made considering medical specialties and areas of medical practice. Courses offered under the names of subspecialties, as well as diagnostic and therapeutic procedures inherent to medical practice, were approximated, preferably considering their respective specialties and areas of practice. Third, free and extension courses, as well as specialization courses that are part of government programs, may not have been reported to the e-MEC by the institutions offering them. The study also failed to analyze the curriculum, content, and quality of the courses.

## CONCLUSION

LSPG courses in medicine in Brazil constitute a heterogeneous universe, offered in different medical areas and varying widely in terms of location, teaching method, workload, number of places, duration, and price. This dispersion reflects the fragility of LSPG regulation and draws attention to the need for the accreditation, certification, and quality assurance of LSPG courses in medicine.

## Data Availability

The datasets generated and/or analyzed during the current study are available from the corresponding author upon reasonable request.
